# A novel nomogram for adult primary perihilar cholangiocarcinoma and considerations concerning lymph node dissection

**DOI:** 10.3389/fsurg.2022.965401

**Published:** 2023-01-06

**Authors:** Qi Zhang, Zehan Liu, Shuangqing Liu, Ming Wang, Xinye Li, Jing Xun, Xiangyu Wang, Qin Yang, Ximo Wang, Dapeng Zhang

**Affiliations:** ^1^ Tianjin Key Laboratory of Acute Abdomen Disease Associated Organ Injury and ITCWM Repair, Tianjin Nankai Hospital, Tianjin Medical University, Tianjin, China; ^2^ Integrated Chinese and Western Medicine Hospital, Tianjin University, Tianjin, China; ^3^ Department of General Surgery, The Third People’s Hospital of Chengdu, Affiliated Hospital of Southwest Jiaotong University & The Second Affiliated Hospital of Chengdu, Chongqing Medical University, Chengdu, China; ^4^ Department of Thoracic Surgery, Sir Run Run Shaw Hospital, School of Medicine, Zhejiang University, Hangzhou, China; ^5^ Academy of Medical Engineering and Translational Medicine, Tianjin University, Tianjin, China

**Keywords:** perihilar cholangiocarcinoma, nomogram, overall survival, lymph node dissection, SEER

## Abstract

**Objective:**

To construct a reliable nomogram available online to predict the postoperative survival of patients with perihilar cholangiocarcinoma.

**Methods:**

Data from 1808 patients diagnosed with perihilar cholangiocarcinoma between 2004 and 2015 were extracted from the National Cancer Institute Surveillance, Epidemiology, and End Results (SEER) database. They were randomly divided into training and validation sets. The nomogram was established by machine learning and Cox model. The discriminant ability and prediction accuracy of the nomogram were evaluated by concordance index (C-index), receiver operator characteristic (ROC) curve and calibration curve. Kaplan-Meier curves show the prognostic value of the associated risk factors and classification system.

**Results:**

Machine learning and multivariate Cox risk regression model showed that sex, age, tumor differentiation, primary tumor stage(T), lymph node metastasis(N), TNM stage, surgery, radiation, chemotherapy, lymph node dissection were associated with the prognosis of perihilar cholangiocarcinoma patients relevant factors (*P* < 0.05). A novel nomogram was established. The calibration plots, C-index and ROC curve for predictions of the 1-, 3-, and 5-year OS were in excellent agreement. In patients with stage T1 and N0 perihilar cholangiocarcinoma, the prognosis of ≥4 lymph nodes dissected was better than that of 1- 3 lymph nodes dissected (*P* < 0.01).

**Conclusion:**

The nomogram prognostic prediction model can provide a reference for evaluating the prognosis and survival rate of patients with perihilar cholangiocarcinoma. Patients with stage T1 and N0 perihilar cholangiocarcinoma have more benefits by increasing the number of lymph node dissection.

## Introduction

Cholangiocarcinoma originates from the epithelium lining of the biliary tree and is classified into intrahepatic cholangiocarcinoma (iCCA, 10%–20%) and extrahepatic cholangiocarcinoma (eCCA), which is further stratified into perihilar cholangiocarcinoma (pCCA, 50%–60%) and distal cholangiocarcinoma (dCCA, 20%–30%) (1–3). In recent years, the incidence and death rate of bile duct cancer are increasing, accounting for about 3% of gastrointestinal malignant tumors and 15% of primary liver cancer (4). Cholangiocarcinomas of different sites have their own characteristics in clinical presentation and, therefore, differ in diagnosis and treatment. PCCA arise from the common hepatic duct, the left and right hepatic ducts and their confluence. It is one of the most common cholangiocarcinomas with insidious onset, rapid progression and asymptomatic. The incidence of pCCA is increasing stably, but the prognosis is generally poor. Surgical resection is the mainstay of potentially curative treatment for pCCA, but less than 40% of patients present with early-stage disease that can be treated with radical resection or liver transplantation (5, 6). PCCA is an aggressive disease and patients have a poor prognosis. Despite the development of multidisciplinary treatments, including surgery, chemotherapy, radiotherapy, immunotherapy and chemoradiotherapy, the prognosis of patients with pCCA remains unfavorable (7, 8). Due to the lack of effective treatments and prognostic indicators, the 5-year overall survival (OS) rate of pCCA is 15%–40% (9, 10). The factors influencing the prognosis of patients with pCCA are therefore extremely important, as well as providing corresponding interventions for patients with different risk levels.

Nomograms and machine learning are widely used statistical tools to estimate the prognosis of individuals and provide more individualized outcome predictions based on integrating diverse biological and clinical variables (11–13). The nomogram is a graphical representation of Logistic regression or Cox regression, which can be used to predict the probability of survival time of individual patients, with high accuracy and good clinical practicability. As an integrative and visualized predictive model, nomogram has been developed in a variety of cancers (14–16). Of note, the establishment of nomograms integrating conventional factors for pCCA has been noted in few studies. Based on the National Cancer Institute: Surveillance, Epidemiology, and End Results (SEER) database, this study retrospectively analyzed the data of pCCA patients, explored the prognostic factors and constructed nomograms (17, 18). Thus, we show the extent to which each clinical variable can influence the prognosis of patients with pCCA. In terms of clinical diagnosis and treatment, an individualized prognosis may serve as a reference.

## Materials and Methods

### Recruitment of patients from SEER database

**Figure 1:**
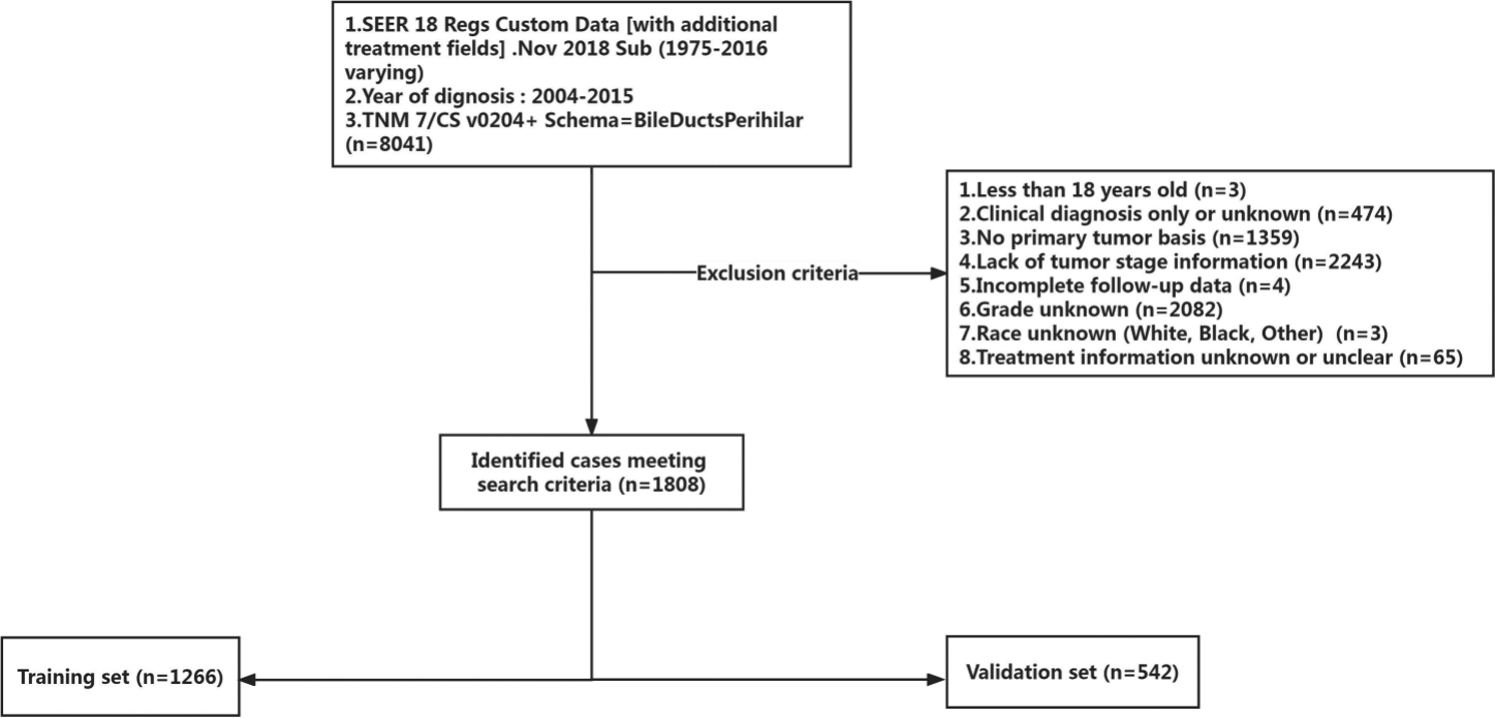
The inclusion and exclusion criteria flowchart of recruited patients in the SEER database. A total of 1,808 patients were included, and these patients were randomly divided into a training set and a validation set.

This study utilized a retrospective cohort design. We extracted the information of patients diagnosed with pCCA (TNM 7/CS v0204+ Schema = BileDuctsPerihilar) between 2004 and 2015 from the SEER database *via* SEER—stat software (SEER*Stat 8.3.9.2). The exclusion criteria were: (1) less than 18 years old; (2) clinical diagnosis only or unknown; (3) no primary tumor basis and more than one primary tumor; (4) lack of tumor stage information; (5) incomplete follow-up data; (6) grade unknown; (7) race unknown (8) treatment information unknown or unclear. A total of 1,808 patients were included, and these patients were randomly divided into a training set (1,266 of 1,808 cases, 70%) and a validation set (542 of 1,808 cases, 30%) according to computer-generated random numbers. The study design is described in a flow chart in Figure 1.

### Clinical variables extracted for analysis

The variables of each patient included clinical and demographic data: age, sex, race, differentiation grade, the American Joint Committee on Cancer (AJCC) TNM stage (including T, N, and M stages), radiation, chemotherapy, surgery, and lymph node dissection. The overall survival (OS) was utilized as the patient outcome measure.

### Statistical methods

Data analysis was performed by R software 4.1.3 (R Foundation for Statistical Computing, Vienna, Austria, www.r-project.org). Statistically, for categorical variables, the chi-square test or continuity-corrected chi-square test was used. Cox proportional hazards model were used for univariate and multivariate analysis. Nomograms were constructed using statistically significant variables in multivariate analysis. Nomogram was visualized using the Sangerbox tools, a free online platform for data analysis (http://vip.sangerbox.com/) (19). Survival analysis was performed using the Kaplan—Meier curves and compared by the Log-Rank test. The forestplot was plotted by http://www.bioinformatics.com.cn/en, an online platform for data analysis and visualization. The accuracy of the nomogram was judged by the Harrell's concordance index (C-index). The calibration of the nomogram was assessed using calibration curves. Specificity and sensitivity were evaluated by the receiver operating characteristics curve(AUC-ROC) (20). External validation was done *via* a validation set. *P *< 0.05 means the difference is statistically significant.

## Results

### Basic patient information

**Figure 2:**
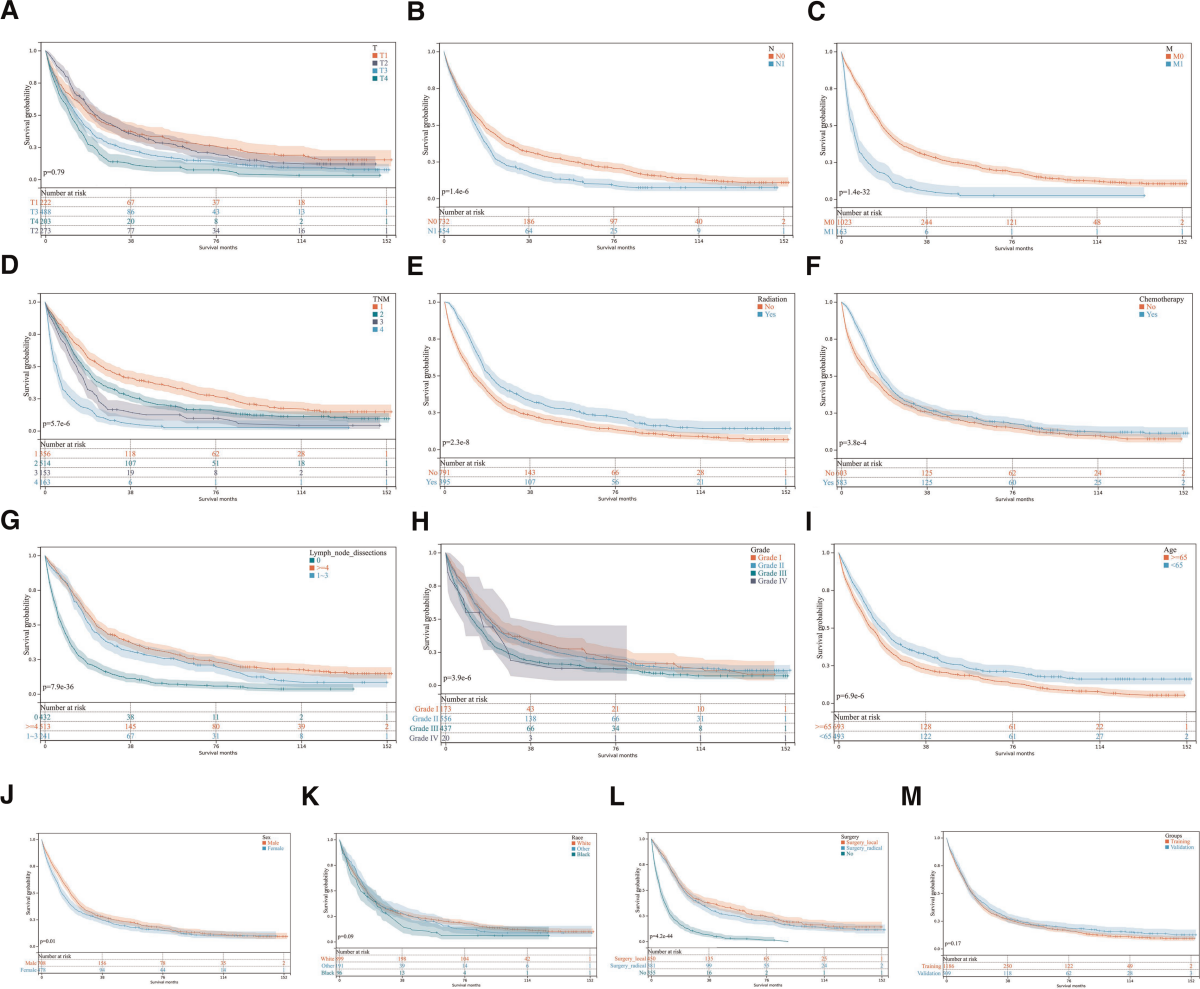
Kaplan-Meier curves for OS. (**A–L**) KM curves show univariate analysis of survival based on different clinical variables. (**A**) T, tumor stage; (**B**) N stage, lymph node metastasis stage; (**C**) M stage, distant metastasis stage; (**D**) TNM stage, Tumor-Node-Metastasis stage (**E**) Radiotherapy; (**F**) Chemotherapy; (**G**) Lymph node dissection; (**H**) Tumor differentiation grade; (**I**) Age; (**J**) Sex; (**K**) Race; (**L**) Surgery method. (**M**) Kaplan-Meier survival analysis in training and validation sets.

A total of 1,808 participants were randomly split into the training set (1,266 cases) and the validation set (542 cases) using a ratio of 7:3. The median survival time of both groups was 13 months, *P *= 0.17, Figure 2M. The basic information of each patient was recorded. Demographic and clinical data at baseline evaluation are shown in Table 1. There were no significant differences in age, sex, race, differentiation grade, TNM stage, or treatment methods between the two groups (*P *> 0.05). This suggested that characteristics of the patients in two sets were similar. There were no differences in the baseline characteristics of the training set and internal validation set.

**Table 1:** Baseline demographic and basic clinical characteristics of patients with pCCA (%).

Demographics and basic clinical characteristics	Training group (*n* = 1266)	Validation group (*n* = 542)	Statistical value	*P* ^a^	Demographics and basic clinical characteristics	Training group (*n* = 1266)	Validation group (*n* = 542)	Statistical value	*P* ^a^
Age					N				
<65	514 (40.6)	203 (37.5)	1.57	0.210	N0	784 (61.9)	326 (60.1)	0.51	0.476
≥65	752 (59.4)	339 (62.5)			N1	482 (38.1)	216 (39.9)		
Sex					M				
Male	763 (60.3)	301 (55.5)	3.51	0.610	M0	1,082 (85.5)	470 (86.7)	0.49	0.485
Female	503 (39.7)	241 (44.5)			M1	184 (14.5)	72 (13.3)		
Race					Lymph node dissection				
White	956 (75.5)	419 (77.3)	0.68	0.713	0	486 (38.4)	201 (37.1)	3.53	0.171
Black	102 (8.1)	41 (7.6)			1–3	250 (19.7)	91 (16.8)		
Other	208 (16.4)	82 (15.1)			≥4	530 (41.9)	250 (46.1)		
Differentiation grade^b^					Surgery				
G1	190 (15.0)	85 (15.7)	0.61	0.895	No	404 (31.9)	167 (30.8)	3.02	0.220
G2	578 (45.7)	238 (43.9)			Surgery_local	472 (37.3)	186 (34.3)		
G3	476 (37.6)	208 (38.4)			Surgery_radical	390 (30.8)	189 (34.9)		
G4	22 (1.7)	11 (2.0)			Radiation				
TNM					Yes	371 (29.3)	146 (26.9)	1.04	0.307
I	378 (29.9)	155 (28.6)	6.04	0.110	No	895 (70.7)	396 (73.1)		
II	535 (42.3)	259 (47.8)			Chemotherapy				
III	169 (13.3)	56 (10.3)			Yes	548 (43.3)	250 (46.1)	0.38	0.540
IV	184 (14.5)	72 (13.3)			No	682 (53.9)	292 (53.9)		
T									
T1	248 (19.6)	114 (21.0)	3.29	0.349					
T2	282 (22.3)	114 (21.0)							
T3	512 (40.4)	234 (43.2)							
T4	224 (17.7)	80 (14.8)							

^a^ *χ*^2^ test.

^b^ Differentiation grade: G1, well differentiated; G2, moderately differentiated; G3, poorly differentiated; G4, undifferentiated.

This study included 1,064 males (58.9%) and 744 females (41.1%), of which 717 (39.7%) patients were ≥65 years old at diagnosis. A total of 1,237 patients (68.4%) received surgical treatment, including radical surgery in 579 (32.0%) patients and local treatment in 658 (36.4%) patients. According to the 6th edition of AJCC staging, 1,142 (63.2%) patients had pT1-pT2 stage, 758 (41.9%) patients had lymph node metastasis (pN1), and 256 (14.2%) patients had distant metastasis (pM1). Among patients with complete clinical data, the majority of patients had medium-low differentiation tumors (83.0%, 1,500/1,808). The overall survival time of patients was 0–155 months, the median survival time was 17 months, and the 1-year OS, 3-year OS, and 5-year OS were 59.3%, 28.0%, and 20.2%, respectively. The median OS was 22 months for patients who underwent surgery and only 6 months for the other patients (*P* < 0.01).

### Analysis of prognostic factors

Univariate and multivariate analyses of prognostic factors of 1,266 pCCA patients in the training set are shown in Table 2. Univariate analysis showed that: sex, age, tumor differentiation, primary tumor stage(T), lymph node metastasis(N), distant metastasis(M), TNM stage, surgery, radiation, chemotherapy and lymph node dissection were associated with the prognosis of pCCA patients relevant factors (*P* < 0.05). The Kaplan-Meier curves were shown in Figures 2A–L. The KM survival analysis is consistent with the univariate Cox analysis.

**Table 2:** Univariate and multivariate Cox analysis of clinical characteristics of patients with pCCA in training set.

Variables	Univariate analysis		Multivariate analysis	
	HR (95%CI)	*P*	HR (95%CI)	*P*
Sex				
Male vs. Female	0.850 (0.748–0.966)	0.0130	0.994 (0.872–1.133)	0.9330
Age				
≥65 vs. <65	1.336 (1.174–1.520)	0.0000	1.254 (1.099–1.431)	0.0010
Race				
Black vs. White	1.259 (1.003–1.581)	0.0470		
Other vs. White	1.015 (0.854 –1.207)	0.8580		
Differentiation grade				
Grade II vs. Grade I	1.050 (0.867–1.273)	0.6120	1.280 (1.051–1.559)	0.0140
Grade III + IV vs. Grade I	1.447 (1.190– 1.759)	0.0000	1.489 (1.218–1.821)	0.0000
T				
T2 vs. T1	1.005 (0.820–1.232)	0.9590	1.287 (1.046–1.584)	0.0171
T3 vs. T1	1.428 (1.192– 1.712)	0.0000	1.370 (1.065–1.763)	0.0145
T4 vs. T1	1.913 (1.548–2.363)	0.0000	1.199 (0.837–1.717)	0.3221
N				
N1 vs. N0	1.363 (1.198–1.550)	0.0000	1.467 (1.257–1.712)	0.0000
M				
M1 vs. M0	2.697 (2.261–3.216)	0.0000	1.444 (1.183–1.762)	0.0000
TNM Stage				
II vs. I	1.368 (1.172– 1.597)	0.0004		
III vs. I	1.843 (1.496–2.269)	0.0000		
IV vs.	3.475 (2.833–4.263)	0.0000		
Surgery				
Surgery_local vs. No	0.306 (0.261–0.358)	0.0000	0.494 (0.392–0.621)	0.0000
Surgery_radical vs. No	0.336 (0.286–0.395)	0.0000	0.509 (0.394–0.659)	0.0000
Radiation				
Yes vs. No	0.686 (0.599–0.785)	0.0000	0.892 (0.760–1.047)	0.1640
Chemotherapy				
Yes vs. No	0.800 (0.705–0.908)	0.0010	0.644 (0.552–0.750)	0.0000
Lymph node dissection				
1–3 vs. 0	0.473 (0.398–0.562)	0.0000	0.661 (0.525–0.832)	0.0000
≥4 vs. 0	0.385 (0.335–0.441)	0.0000	0.5112 (0.408–0.641)	0.0000

**Figure 3:**
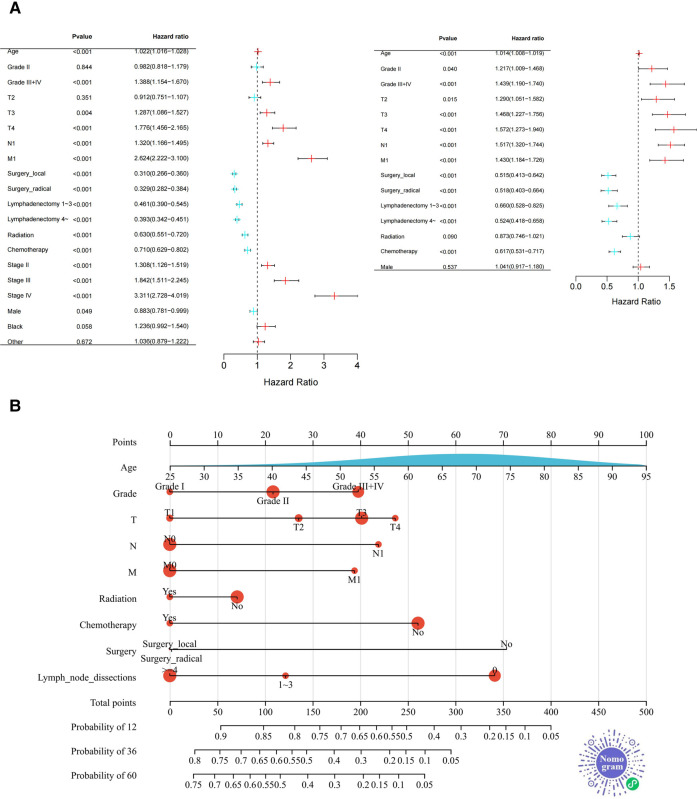
Forest plot and nomogram for training set. (**A**) The Forest Plot shows the results of Cox proportional Hazard regression analysis (Left: univariate analysis; right: multivariate analysis. (**B**) The novel nomogram was constructed based on Independent prognostic factors for predicting 1-, 3-, or 5-year survival rates in pCCA. As shown above, the 9 variables correspond to the upper level subscales, and the total score of each variable down corresponds to the 1, 3, and 5 year overall survival associated with the prediction. The area of the red dots represents the number of samples and the distribution of age (a continuous variable) is presented as the area under the blue curve. Two-dimensional code that makes the nomogram accessible in WeChat.

Multivariate analysis showed that the primary tumor stage(T), lymph node metastasis(N), distant metastasis(M), surgery, chemotherapy, and lymph node dissection were independent risk factors for the prognosis of pCCA patients (*P *< 0.05). To make the presentation clearer, the results of the multivariate analysis were shown in the form of a forest diagram in Figure 3A. Age was modelled as a continuous variable in the forest map.

### Construction and publication of the Nomogram

Independent prognostic factors based on multivariate analysis for the training set were applied to establish predictive nomograms, Figure 3B. According to the different classifications of each factor, the score of each item can be calculated by projecting upward to the small scale. The total score is obtained by summing each score and the patient's 1, 3- and 5-year overall survival rates can be obtained by projecting downward from the total scale. Different from the common nomogram, we draw a novel nomogram that displays the sample distribution and reference line, which is convenient for the calculation of the total score. For the convenience of doctors (especially Chinese doctors) in clinical practice, we will publish the nomogram in the WeChat miniprograms platform later and the two-dimensional code to enter the miniprograms is shown in Figure 3B.

### Nomogram validation

**Figure 4:**
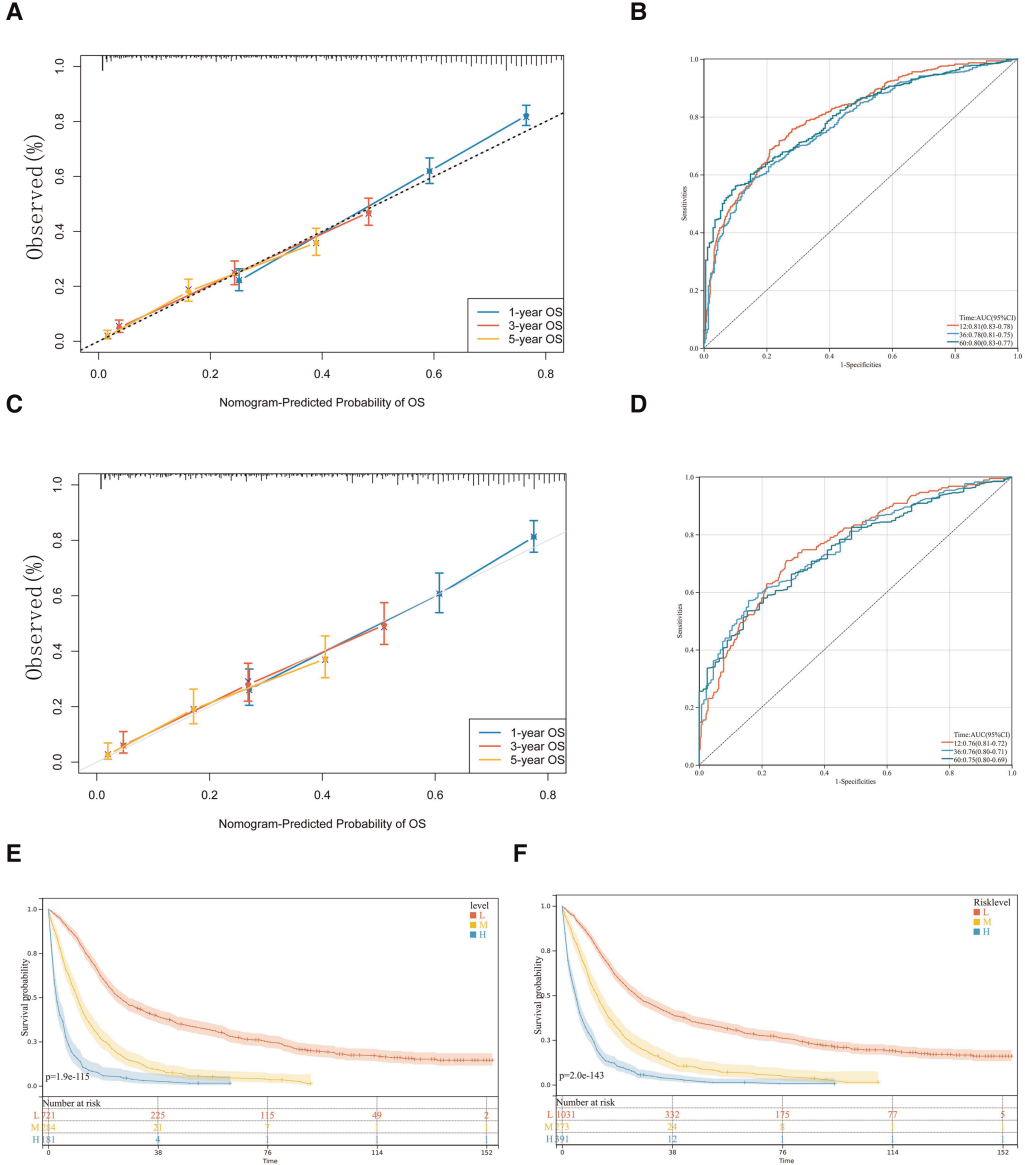
Calibration slope and receiving operating characteristic (ROC) curve of our model. The 1-year, 3-year and 5-year calibration curves for the training set (**A**) and validation set (**C**) are also close to the ideal straight line with a slope of 1. In ROC curves of training set (**B**) and validation set (**D**), nomogram has high accuracy in predicting the prognosis of patients with pCCA. Kaplan-Meier curves for the OS of pCCA patients in different level of risk in training set (**E**) and total set (**F**) according to risk-score.

The nomogram was verified internally and externally by the Bootstrap method, the ROC curve was drawn for the model and the calibration curve was established. Meanwhile, the C-index was used in two groups for validation. The C-index of the nomogram was 0.726 (95% CI, 0.711–0.741) in the training set and 0.703 (95% CI, 0.679–0.728) in the validation set, which demonstrated its outstanding prediction accuracy. The calibration curves of 1-year, 3-year and 5-year survival rates are all close to the ideal straight line with a slope of 1, Figure 4A. In the training set, the area under the ROC curve (AUC) for 1-year, 3-year and 5-year survival was 0.81, 0.78 and 0.80, respectively, also indicating that the model has a good predictive value, Figure 4B. The calibration curves were also close to the ideal straight line with a slope of 1, 4C. The nomogram had good accuracy in both the training set and validation set. The validation set AUC for survival was 0.76, 0.76 and 0.75, respectively, Figure 4D.

**Figure 5:**
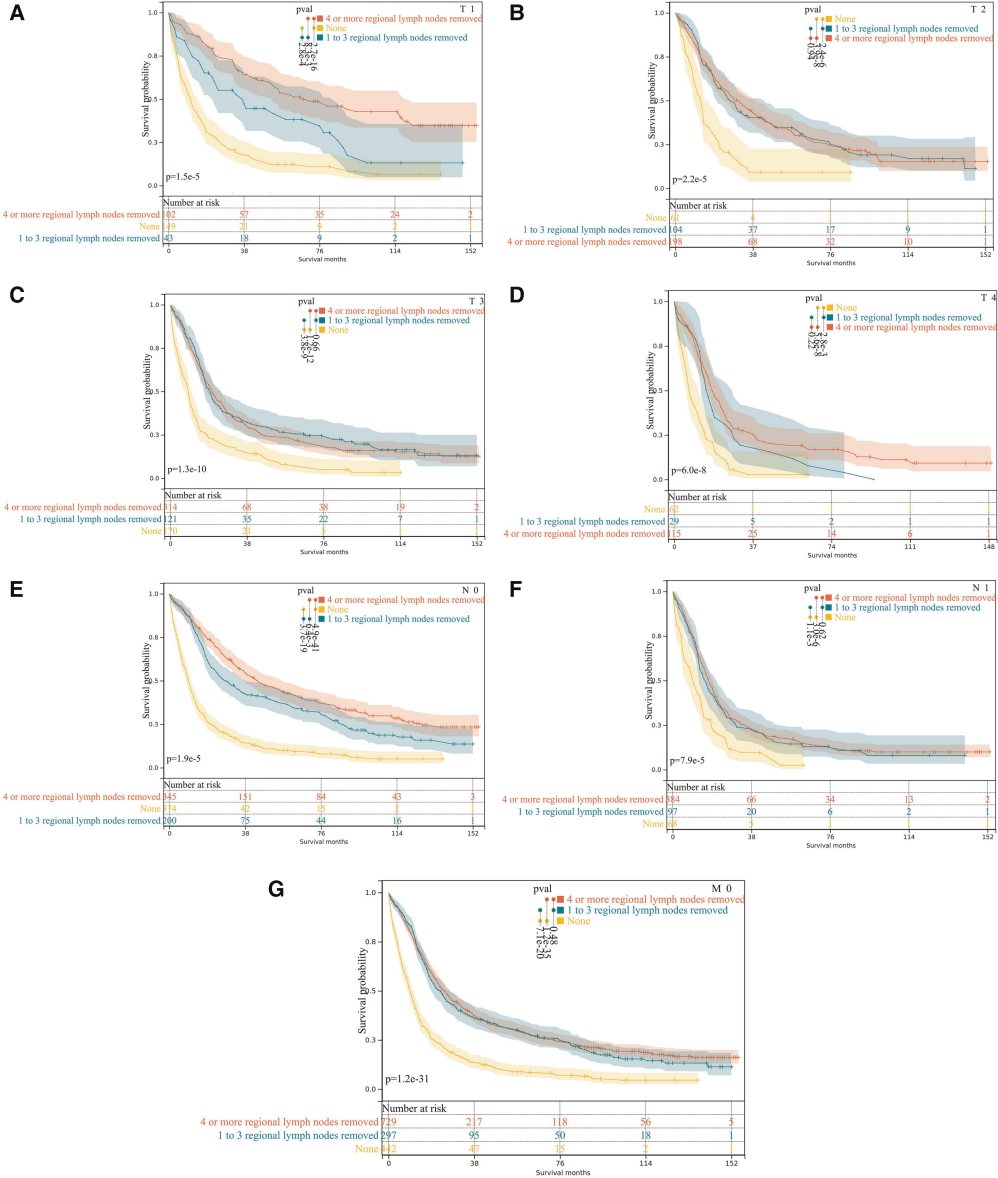
Subgroup analysis of lymph node dissection in M0 pCCA patients by Kaplan-Meier curves. (**A–D**) T1-T4; (**E,F**) N0 and N1; (**G**) the subgroup of M0.

We trichotomized the total scores calculated from nomograms by X-tile software to achieve the maximum difference in survival outcomes (21). Specifically, the software achieved the maximum difference in survival outcome by selecting the highest chi-square value in the survival analysis, indicating that the total score calculated from the nomogram using the X-tile software was divided into three subgroups of patients with low (<220), medium (220 to 309), and high risk (>309). The significance of the prognostic differences between the samples of different groups was evaluated by the Log-rank test. Finally, we observed the significant prognostic differences both in training and total sets according to risk-score, Figures 5E,F.

### Subgroup analysis

The analysis found that the prognosis of patients with dissection of lymph nodes is better than that of patients without dissection. Next, we performed stratified analyses for associations between OS and the dissection of lymph nodes. However, the prognosis of M0 patients with dissection number ≥4 lymph nodes is not superior to that of patients with 1–3 lymph nodes dissection(*P *= 0.48), Figure 5G. Moreover, the stratified analysis showed that among patients with T1 pCCA after surgery, the number of lymph nodes dissected ≥4 lymph nodes were more profitable than those with 1–3 lymph nodes, Figure 5A. However, in patients with T2—T4 pCCA after surgery, there was no statistically significant difference in the prognosis between the number of lymph nodes dissected ≥4 and those with 1–3 lymph nodes, Figures 5B–D. Similarly, in patients with stage N0, the number of lymph nodes dissected ≥4 lymph nodes was more beneficial than the dissection of 1–3 lymph nodes, Figure 5E. In N1 stage patients, there was no statistically significant difference in prognosis between those with ≥4 lymph nodes dissected and those with 1–3 lymph nodes, Figure 5F.

## Discussion

Prognostic evaluation of tumor patients has important clinical significance in the process of treatment, monitoring and follow-up. Due to pCCA low incidence and rarity (It only accounts for less than 2% of digestive system tumors), there are few studies related to the prognostic factors about pCCA and most of the current studies are single-center or relatively small sample size (22, 23). In this study, based on the public data of the SEER database, a nomogram model for prognosis prediction of pCCA patients was constructed, which has a high accuracy rate. We use a novel nomogram, which not only facilitates the calculation of scores but also provides an overview of the distribution of the samples. In the future, it will be released through a miniprogram, so that the research results can be transformed and popularized. In recent years, nomograms have flourished in the medical field, and a large number of nomogram-related studies are published every year. However, there is no suitable tool to help clinicians to apply these nomograms efficiently in clinical practice. The Nomogram Platform is an online application for a wide range of high-quality medical Nomograms. The platform is designed to facilitate both the promotion of nomograms by developers and the use of nomograms by clinicians. In the future, we will publish the nomogram of this study on the miniprogram, the entrance of which is shown in Figure 3B.

The prognosis of elderly cancer patients cannot be ignored. There is a lot of evidence and theories to explain the reduced survival of elderly patients, which mainly includes the bias of treatment options to the elderly, resulting in inadequate surgery, chemotherapy, and less participation in clinical trials. As well as poor nutrition and poorer immune monitoring in the elderly (13, 24, 25). The results of this study suggest that ≥65 years of age is an independent factor influencing the prediction of OS, which is consistent with the above explanations.

Lymph node metastasis is the most common way of metastasis in pCCA, so lymph node dissection is an important part of radical surgery in pCCA. Adequate dissection of lymph nodes has important clinical value for accurate staging of pCCA and determination of adjuvant treatment strategies but the number and extent of lymph node dissection are still controversial. The criteria for the number of detected lymph nodes are not indicated in the Hepatobiliary Cancers, Version 2.2021, NCCN Clinical Practice Guidelines in Oncology (26, 27). However, the Chinese Society of Clinical Oncology (CSCO) guidelines for the diagnosis and treatment of cholangiocarcinoma point out that the number of lymph nodes detected in pCCA should be ≥6 as much as possible (28). Most scholars believe that in addition to improving the accuracy of staging, extended lymph node dissection is meaningless. Because expanded dissection not only increased complications but also showed no significant benefit in OS (29). In this study, lymph node dissection can benefit patients with any T and N stages. In terms of the number of dissected lymph nodes, a stratified analysis of T and N staging found that in T1 and N0 pCCA patients with M0 stage, patients with ≥4 lymph nodes dissected had greater benefit than those with 1 to 3 lymph nodes dissected. However, in T2, T3, T4, and N1 stage patients, there was no significant difference in prognosis between the two types of lymph node dissection numbers. This result was frustrating for surgeons, suggesting that for lymph node metastases, surgery alone cannot further improve outcomes. One study showed that the total number of lymph nodes removed affects overall survival in N0 patients, with the removal of more than 5 lymph nodes being the minimum number required for adequate staging. They found no significant difference in 5-year OS in N1 patients who had 1 to 5 lymph nodes removed and those who had more lymph nodes removed (23). Studies also believe that for N1 patients after liver resection, the lymph nodes rate >0.20–0.27, rather than the number of lymph nodes dissected, is the only independent risk factor for OS (30, 31). These results were generally consistent with our study.

We noted that patients with extra-regional lymph node involvement were classified as M0 according to the AJCC 6th edition criteria, which led to an underestimation of the M1 patient population. Of note, the AJCC 8th edition revision focuses on staging regional lymph node involvement based on the number of positive lymph nodes, N1 < 4 lymph nodes involved and N2 ≥ 4 involved (32, 33). Also, according to 8th edition, relatively distant lymph node metastases such as para-aortic, para-inferior vena cava, superior mesenteric artery, and truncal artery lymph nodes should be categorized as M1, not N+ (34). Generally, retrospective TNM staging was no longer possible with a large number of specimens due to the omission of critical information from pathology reports (35). Therefore, it is recommended to popularize tabular pathology reports to facilitate future retrospective studies. We also considered using AJCC 7th edition or recoding to AJCC 8th edition, but encountered the following insurmountable problems during implementation: (1): the 7th edition staging cannot be accurately recoded to the 8th edition staging. In the 7th edition, N2 can indeed be recoded as M1 in the 8th edition, but N2 in the 8th edition may be implied in N1 in the 7th edition, and this part of the data can no longer be recoded (e.g., in 7th edition, some N1 patients had >4 regional lymph node metastases but mainly limited around the perihilar region and these patients should be classified as N2 in the 8th edition. We had considered recoding the staging data but lacked accurate regional lymph node information in the SEER database.). (2), Due to the relatively small incidence of pCCA and the limited use of 8th edition, the sample size of the 8th edition in the SEER database is far from sufficient for modeling. We expect that the data from the 8th edition in SEER will lead to a better modeling base after accumulation in the coming years.

Surgery, whether radical or non-radical, is critical to the long-term survival of patients with pCCA. Our Cox results support this opinion. Owing to the special anatomical location and biological behavior of pCCA, in addition to the axial resection margin of the bile duct, the lateral resection margin of the hepatic hilar region and the parenchymal resection margin should also be considered (36). Under this criterion, numerous of their R1 patients may be falsely classified as R0, and the 5-year OS rate with a true R0 resection can be as high as 67% (10, 37). Here, we hope that more readers will become aware of the lack of actual implementation rates of radical pCCA surgery. In addition, we did not include data on liver transplantation. In fact, this is also a type of radical surgery. Surgery is usually the preferred treatment option, but liver transplantation after neoadjuvant chemotherapy is also an option for a minority of patients with pCCA. Regardless of the fact that there is controversy over whether pCCA patients should undergo liver transplantation, the current mainstream view is that patients who have been carefully screened and preoperative preparation can often benefit from liver transplantation (38–41).

PCCA prognosis is usually poor due to its late manifestations and its relative resistance to current chemotherapy and radiotherapy regimens. The current clinically used radiotherapy modalities are mainly external beam radiotherapy alone and external beam radiotherapy combined with intracavitary radiotherapy. For extrahepatic cholangiocarcinoma, a combination of chemotherapy and radiotherapy is recommended, mainly including postoperative adjuvant chemoradiotherapy, preoperative neoadjuvant chemoradiotherapy and palliative radiotherapy for unresectable and metastatic cholangiocarcinoma (42–45). Of these, the strongest recommendation of evidence grade is postoperative adjuvant chemoradiotherapy (46, 47). The positive effects of chemotherapy and radiotherapy were equally confirmed in the study. Based on our results, chemotherapy appears to provide greater benefit than radiotherapy. However, based on the limited data provided by SEER, we were unable to analyze the clinical value of the combination of radiotherapy and chemotherapy, as well as neoadjuvant therapy.

There are some imperfections in this study, such as the number and location of lymph node dissection. First, in this study, the number of lymph nodes dissected was only vaguely classified (0, 1–3, ≥4) according to SEER, so the analysis conclusion was not accurate enough. In fact, we used a relatively superficial univariate analysis that needs to be supported by additional studies. Second, the SEER database lacks Bismuth-Corlette classification, Blumgart staging, CA199/CEA levels, liver transplantation, novel drug loading methods, targeted and immunotherapy data. Patients with pCCA may profit from these new therapeutic strategies (41, 48–50). Adding such data when constructing the nomogram will make the prediction results more accurate and individualized. Third, due to the limited sample size, we did not include the sequence of chemoradiotherapy and surgery into the statistics, so we did not accurately assess the value of neoadjuvant chemoradiotherapy for pCCA. Finally, although nomograms have achieved good accuracy, prospective studies are warranted to confirm the reliability of the nomograms.

## Conclusion

In conclusion, age, tumor differentiation, T stage, N stage, M stage, surgery, chemotherapy and the number of surgical lymph node dissections are independent risk factors that affect the prognosis of patients with pCCA. The establishment of a nomogram prediction model can predict the prognosis individually, intuitively and accurately. In terms of the number of lymph nodes dissected, there was a prognostic benefit by increasing the number of lymph nodes dissected in patients with stage T1, whereas in patients with stages T2–T4, increasing the number of lymph nodes dissected did not significantly improve the prognosis of the patients.

## Data Availability

The raw data supporting the conclusions of this article will be made available by the authors, without undue reservation.
